# Anatomical Trickery in Acute Cholecystitis: Very Low Cystic Duct Insertion Near the Ampulla of Vater

**DOI:** 10.7759/cureus.96624

**Published:** 2025-11-11

**Authors:** Kyungchul Kim, Dillion Conway, Alfredo Noches-Garcia

**Affiliations:** 1 Department of General Surgery, Rockingham General Hospital, Perth, AUS

**Keywords:** ampulla of the vater, biliary anatomy variant, endoscopic retrograde cholangiopancreatography (ercp), intraoperative cholangiography, low insertion cystic duct, magnetic resonance cholangiopancreatography (mrcp)

## Abstract

Anatomical variations of the cystic duct are relatively common; however, insertion at or near the ampulla of Vater is exceptionally rare and carries significant implications for surgical and endoscopic management. We report a case of a 69-year-old male who presented with acute gangrenous cholecystitis and biliary sepsis. In our setting, magnetic resonance cholangiopancreatography (MRCP) is not routinely performed for acute cholecystitis, being reserved for stable patients with obstructive liver function tests or suspected choledocholithiasis. Most patients are adequately assessed using ultrasound, and when indicated, computed tomography. Given the patient’s instability and urgent need for source control, preoperative MRCP was not obtained. Intraoperative cholangiography (IOC) was undertaken during laparoscopic cholecystectomy and demonstrated a very low cystic duct insertion at the level of ampulla of Vater. The operation proceeded with careful dissection to achieve the critical view of safety (CVS). Postoperative MRCP, arranged after the acute episode, confirmed the IOC findings. The patient had an uncomplicated recovery and was discharged on postoperative day five. Very low cystic duct insertion increases the risk of bile duct injury and may cause technical challenges during both laparoscopic cholecystectomy and endoscopic retrograde cholangiopancreatography (ERCP). This case highlights the importance of intraoperative biliary mapping in complex or emergency gallbladder surgery, especially when preoperative imaging is not feasible, not indicated, or not accessible, and reinforces the role of IOC in safely identifying rare anatomical variants.

## Introduction

The cystic duct exhibits significant anatomical variability in its course and insertion into the extrahepatic biliary tree. In most individuals, the cystic duct joins the common hepatic duct (CHD) at the mid-portion between the hepatic hilum and the ampulla of Vater [1]. Variants, such as medial insertion, low insertion into the distal common bile duct (CBD), and long parallel coursing, are not uncommon and are seen in up to 10% of cases [2,3]. However, insertion of the cystic duct near the pancreatic head or ampulla of Vater is exceedingly rare, with only a few isolated cases documented in the literature [4,5].

The clinical implications of such an anomaly are significant. If unrecognized, a very low insertion of the cystic duct can lead to misidentification of biliary structures during laparoscopic cholecystectomy, increasing the risk of bile duct injury - a complication associated with high morbidity and litigation [6]. Furthermore, during endoscopic procedures, such as endoscopic retrograde cholangiopancreatography (ERCP), such low insertions may result in inadvertent stenting of cystic duct [5].

Accurate preoperative delineation of biliary anatomy is essential. Magnetic resonance cholangiopancreatography (MRCP) is a valuable, non-invasive tool for detecting variants of cystic duct anatomy, including low insertions [3]. However, in emergency settings, patients with biliary sepsis or acute gangrenous cholecystitis may be too unstable to undergo MRCP, or access to magnetic resonance imaging (MRI) may be limited. Ultrasonography remains the first-line imaging to evaluate the gallbladder and biliary tree, while computed tomography (CT) may be employed to identify complications of cholecystitis. In these situations, intraoperative cholangiography (IOC) becomes indispensable, allowing real-time visualization of biliary tree anatomy and reducing the risk of iatrogenic injury when the anatomy is unclear.

We report a case of acute cholecystitis in a 69-year-old male with biliary sepsis, where an intraoperative cholangiogram revealed an exceptionally low insertion of the cystic duct at the level of the ampulla of Vater, which was later confirmed by MRCP. To our knowledge, such findings remain exceedingly uncommon and add to the limited literature on extremely rare cystic duct variants and highlight how intraoperative cholangiography can decisively alter operative strategy, preventing potentially catastrophic complications.

## Case presentation

A 69-year-old male presented to the emergency department at night with four days of postprandial upper abdominal pain and associated nausea and vomiting. This was associated with fevers and rigors. He denied any recent history of non-steroidal anti-inflammatory medications or alcohol consumption.

The patient had a history of upper gastrointestinal bleeding secondary to a perforated duodenal ulcer in 2020 from *Helicobacter pylori* infection. This was not amenable to endoscopic therapy due to the size of the bleeding vessel, requiring gastroduodenal artery (GDA) embolization. The duodenal ulcer perforation was mostly contained and therefore managed conservatively with intravenous proton pump inhibitor therapy and antibiotics. Other relevant medical history included type II diabetes mellitus.

On physical examination, he was tachycardic with a heart rate of 110 beats per minute, blood pressure of 112/64 mmHg, and febrile at 38.4°C. His abdomen was soft, and there was a right upper quadrant abdominal tenderness with positive Murphy’s sign.

**Figure 1:**
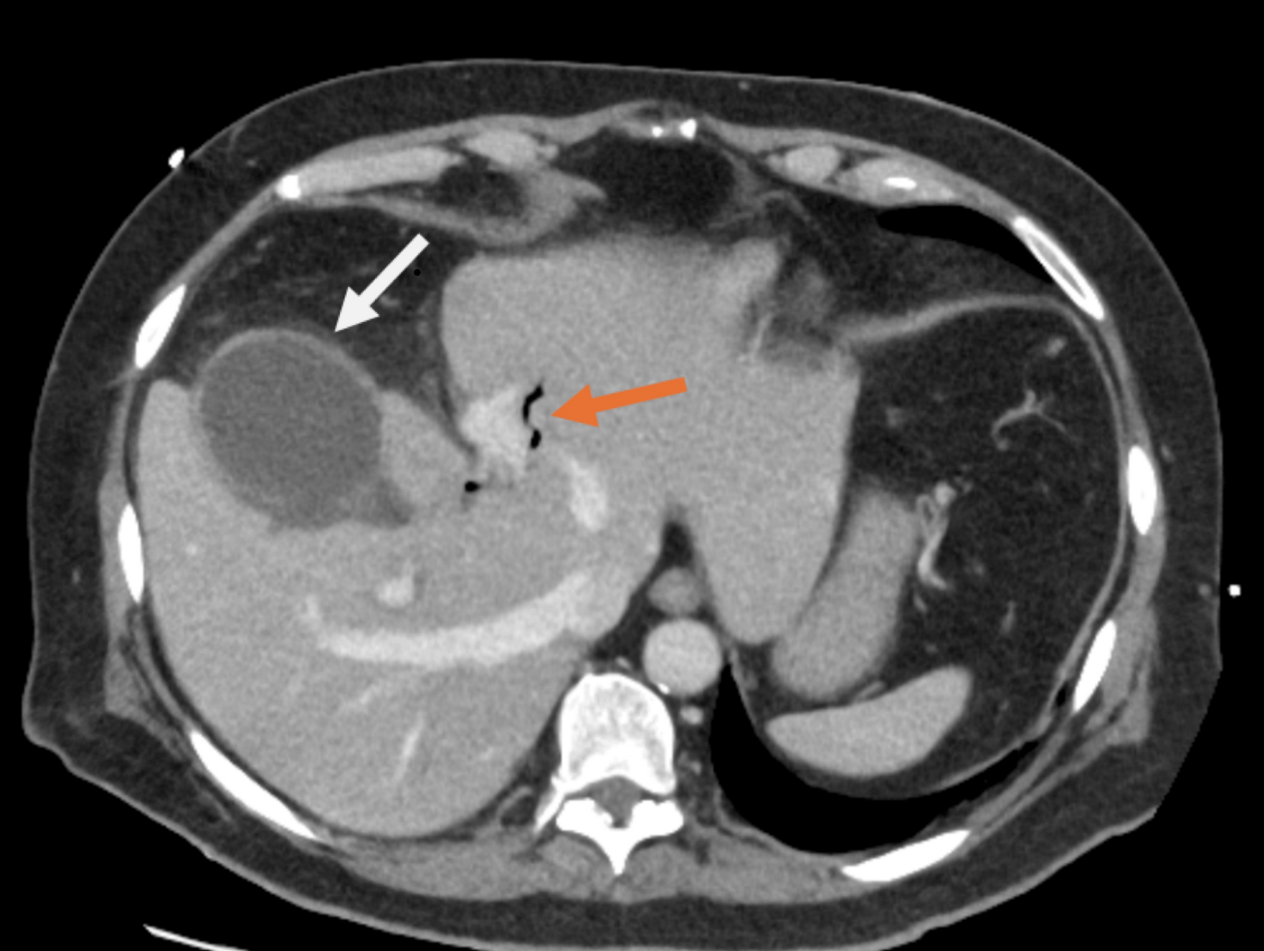
Axial slice of a CT abdomen and pelvis with contrast (portovenous phase). The image shows a distended gallbladder with mural thickening and pericholecystic fluid (white arrow) and a longstanding pneumobilia (orange arrow).

**Figure 2:**
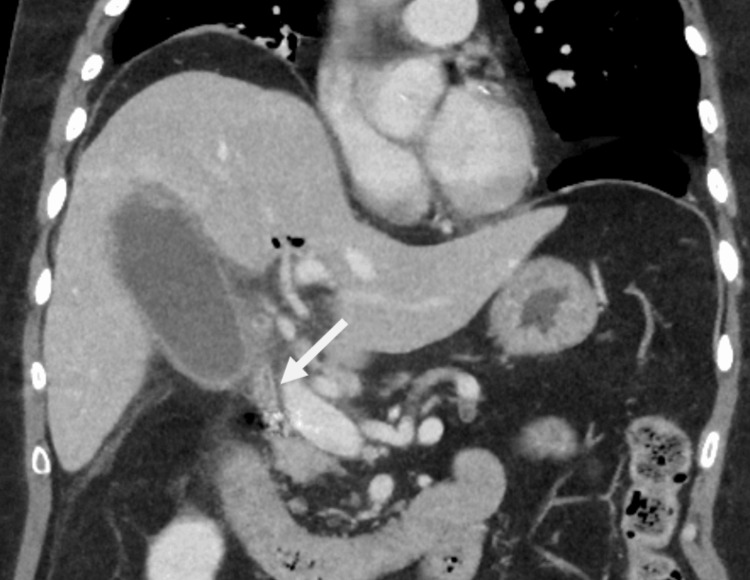
Coronal slice of a CT abdomen and pelvis with contrast (portovenous phase). The image shows a non-dilated CBD (white arrow). CBD: common bile duct

Routine blood investigations demonstrated a significantly elevated C-reactive protein (CRP) of 523 mg/L, white cell count of 5.73×10^9^/L, mildly deranged liver function test (LFT) with total bilirubin of 25 µmol/L, alanine aminotransferase (ALT) of 88 U/L, alkaline phosphatase (ALP) 295 U/L, gamma glutamyl transferase (GGT) 67 U/L, and a normal lipase (Table 1). A CT scan of his abdomen and pelvis with intravenous contrast was performed to investigate for potential complications of biliary pathology with high inflammatory markers, and also due to a lack of ultrasound availability at night. It demonstrated a distended gallbladder with mural thickening and pericholecystic fat stranding without any biliary dilatation or any perforation (Figures 1, 2). There was evidence of previous GDA embolization and a longstanding pneumobilia due to ampullary dysfunction from duodenal perforation back in 2020.

**Table 1:** Preoperative laboratory findings.

Parameters	Preoperative value	Normal range	Units
White blood cells (WBC)	5.73	4.00-11.0	10^9^/L
C-reactive protein (CRP)	523	<5.0	mg/L
Total bilirubin	25	<20.0	µmol/L
Alanine aminotransferase (ALT)	88	<40	U/L
Alkaline phosphatase (ALP)	295	30-110	U/L
Gamma-glutamyl transferase (GGT)	67	<60	U/L
Lipase	22	20-210	U/L

**Figure 3:**
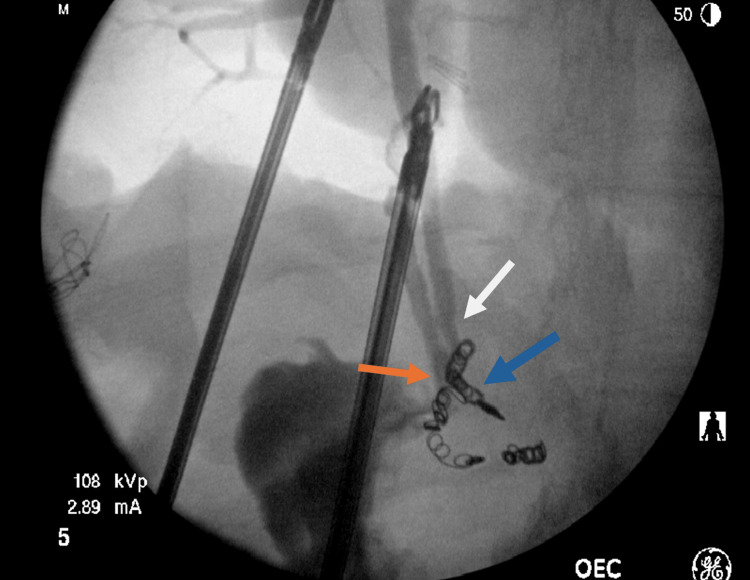
Intraoperative cholangiogram demonstrating cystic duct insertion at the level of ampulla (orange arrow) with contrast flow to duodenum. CBD (white arrow) was seen parallel to the cystic duct. Coils from prior GDA embolization are visible (blue arrow). CBD: common bile duct; GDA: gastroduodenal artery

**Figure 4:**
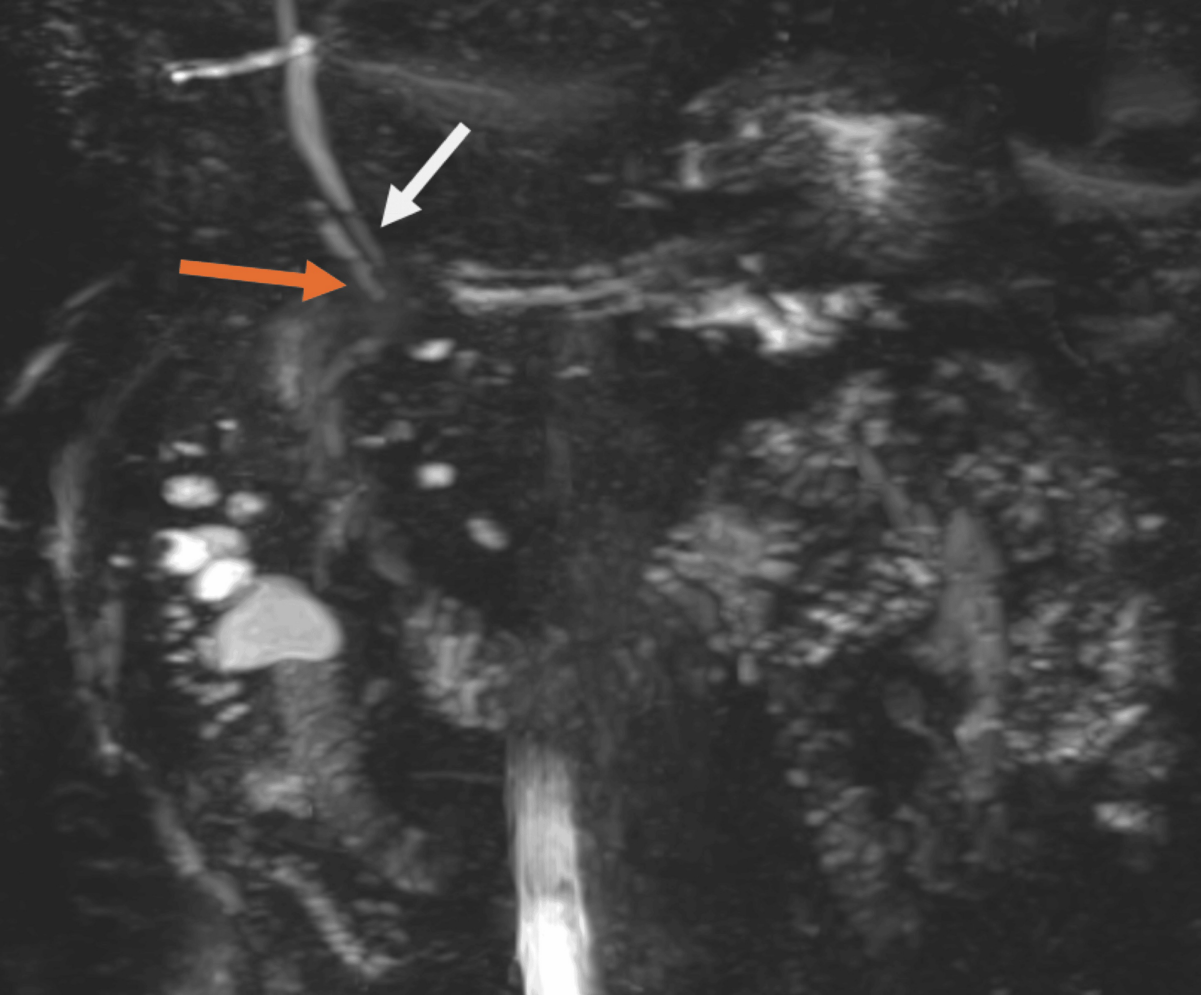
An T2-weighted MRCP SPACE sequence was used to visualize biliary tree. The white arrow indicates the CBD, and the orange arrow demonstrates the cystic duct coursing parallel to the CBD and inserting near the ampulla of Vater. MRCP: magnetic resonance cholangiopancreatography; CBD: common bile duct

Following the investigations, he was diagnosed with acute cholecystitis with biliary sepsis and was admitted to the general surgery unit for further management. Initially, an insulin and dextrose infusion was commenced to prevent euglycemic diabetic ketoacidosis while fasting. He underwent operative management with laparoscopic cholecystectomy and intraoperative cholangiogram the following morning. Intraoperatively, there was evidence of significant inflammatory adhesions with an acute gangrenous necrotic gallbladder. Dissection of Calot’s triangle proved difficult due to significant inflammatory changes. With cautious dissection, a critical view of safety (CVS) was achieved, and an intraoperative cholangiogram was performed, which demonstrated a very low insertion of the cystic duct near the level of the ampulla with contrast flow to the duodenum (Figure 3). Postoperatively, he was admitted to the high dependency unit for 24 h for monitoring and was eventually stepped down to the ward. He was discharged six days post the operation without postoperative complications. In the outpatient setting, an MRCP was subsequently performed, which confirmed the intraoperative cholangiogram findings of a very low cystic duct insertion (Figure 4).

## Discussion

This case describes an exceptionally rare anatomical variant of the biliary tree - a very low insertion of the cystic duct at the level of the ampulla of Vater - discovered intraoperatively during emergent laparoscopic cholecystectomy for acute gangrenous cholecystitis. While cystic duct variations, such as medial or low insertion into the distal common bile duct, occur in up to 10% of cases, an insertion this close to the ampulla is seldom encountered and has been documented in only a handful of case reports [2-5].

In standard anatomy, the cystic duct joins the common hepatic duct well above the ampulla, creating a safe dissection zone in Calot’s triangle. A very low insertion places the junction close to the pancreatic head and duodenal wall. In the presence of inflammation, edema, or adhesions, the convergence point can be obscured, increasing the risk of mistaking the CBD for the cystic duct. Such misidentification is a well-recognized mechanism for major bile duct injury, which carries significant morbidity and long-term sequelae [6].

Limitations of preoperative imaging in emergencies

MRCP is highly sensitive for defining biliary anatomy and reliably depicts cystic duct variants [3]. However, in emergency presentations with sepsis or gangrenous cholecystitis, the urgency of source control often precludes the time needed for advanced imaging. Ultrasound and CT can confirm cholecystitis or complications, but rarely define ductal anatomy unless there is marked biliary dilatation. In such situations, IOC becomes the only real-time mapping tool available.

Role of intraoperative cholangiography

IOC enabled recognition of the variant anatomy in this case, guiding careful dissection and preventing CBD injury. While IOC is not performed routinely in every center in Western Australia, this case illustrates its value in emergency cholecystectomy when anatomy is unclear. More liberal IOC use in inflamed or complex cases may improve safety and warrant further evaluation.

Historically, unclear anatomy often led to early conversion to open cholecystectomy or default subtotal cholecystectomy [7]. Modern laparoscopic practice emphasizes the CVS as the safeguard, supported by selective IOC [8]. However, cases like this invite reconsideration of whether more liberal IOC use in emergency cholecystectomy might prevent catastrophic bile duct injuries. Equally, this case reaffirms the role of alternative dissection strategies, such as fundus-first or subtotal cholecystectomy, when standard CVS is threatened.

Endoscopic considerations

Low cystic duct insertion also has implications for endoscopic retrograde cholangiopancreatography (ERCP). The proximity of cystic duct to the ampulla increases the risk of inadvertent cannulation or stenting, potentially leading to failed drainage or obstruction [5]. Furthermore, low insertion has been associated with a higher prevalence of choledocholithiasis, possibly due to bile stasis [9]. Awareness of this variant is essential for endoscopists, especially in the absence of prior cross-sectional imaging.

## Conclusions

Very low insertion of the cystic duct at the level of the ampulla of Vater represents an exceptionally rare anatomical variant with important implications for both surgical and endoscopic management. In this case, intraoperative cholangiography clearly demonstrated the anomalous course of the cystic duct, allowing intraoperative recognition and precise dissection that averted inadvertent bile duct injury. Subsequent MRCP confirmation validated the intraoperative findings and illustrated the complementary role of postoperative imaging in defining complex anatomy. This case underscores that vigilance for biliary variants, meticulous surgical technique, and the routine use of IOC are essential safeguards for safe cholecystectomy in the acute setting.
